# Cytotoxic Effect of Eudesmanolides Isolated from Flowers of *Tanacetum vulgare s*sp. *siculum*

**DOI:** 10.3390/molecules17078186

**Published:** 2012-07-09

**Authors:** Sergio Rosselli, Maurizio Bruno, Francesco Maria Raimondo, Vivienne Spadaro, Mehmet Varol, Ayşe Tansu Koparal, Antonella Maggio

**Affiliations:** 1 Department of Molecular and Biomolecular Science and Technology (STeMBio), Organic Chemistry Section, Palermo University, Viale delle Scienze, Palermo 90128, Italy; 2 Department of Environmental Biology and Biodiversity, Palermo University, Via Archirafi 38, Palermo 90123, Italy; 3 Department of Biology, Faculty of Science, Anadolu University, Yunusemre Campus, Eskisehir TR 26470, Turkey

**Keywords:** *Tanacetum vulgare*, sesquiterpenes, eudesmanolides, cytotoxic activity

## Abstract

A phytochemical analysis of the dichloromethane extract from the flowers of a subspecies of *Tanacetum vulgare* growing in Sicily was carried out. Five known sesquiterpene lactones with the eudesmane skeleton have been isolated and the cytotoxic activity of these compounds was tested *in vitro* on A549 (human lung carcinoma epithelial-like) and V79379A (Chinese hamster lung fibroblast-like) cells using the tetrazolium salt reduction (MTT) assay. All of tested compounds induced high time- and concentration-dependent cytotoxic effects.

## 1. Introduction

The genus *Tanacetum* comprises about 150 species that are commonly found in Europe and Asia from the Mediterranean to Iran. Some members of this genus are important medicinal plants used over the years in all over the World. Many traditional treatments use *Tanacetum* spp. to heal wounds and ulcers, fever, headache, gastrointestinal diseases. One of the main uses of some species of *Tanacetum* and traditional medicines derived from them, is the treatment of inflammation. *Tanacetum* extracts have been also reported to exhibit antitumor [1], anti-inflammatory [2], antioxidant [3], and antimicrobial activity [4].

*Tanacetum vulgare* (Asteraceae/Compositae, syn. *Chrysanthemum vulgare* L.), known by the common name of Tansy, shows a lot of very interesting and examined pharmacological aspects. It is widely used in folk medicine and the crude toxic Tanaceti flos, described for years in some Western pharmacopoeias, has been employed as a vermifuge, emenagogue [5] and anti-inflammatory treatment [6]. *T. vulgare* shows also remarkable antioxidant properties, mainly due to its phenolic compounds content [7], in fact it is particularly rich in flavonoids [8], along with 3,5-dicaffeoylquinic acid, showing antiviral activity against the herpes simplex viruses HSV-1 and HSV-2 [9].

*T. vulgare* subsp. *siculum* ethanol extract revealed a moderate serotonine release inhibitory activity suggesting its potential use in the symptomatic treatment of migraines [10]. This wide spectrum of activities can be mainly ascribed to the occurrence of sequiterpene lactones (STLs) whose distribution within the plant kingdom reveals a strong concentration in the Asteraceae family representing the major source of STLs’ structural diversity.

With the aim to isolate STLs, we carried out a phytochemical analysis of the flowers of *T. vulgare* subsp. *siculum* and five previously known eudesmanolides were isolated and characterized. In this paper we only report the ^13^C-NMR data of those compounds which was never previously reported in the literature to our knowledge.

There is strong evidence that the cytotoxic activity of STLs is due to the presence of the unsaturated lactone functionality which is highly reactive towards suitable nucleophiles, e.g., sulfhydryl groups of cysteine, by a Michael addition. nevertheless the mechanism of action is not well established. A great number of STLs possess considerable anti-inflammatory activity related to the inhibition of the transcriptor factor NF-κB [11] that plays a pivotal role in the regulation of the cell homeostasis, apoptosis and tumour growth [12] and it was straightforward to assume a link between the known cytotoxic activity of STLs and their NF-κB inhibitory activity. This inhibitory activity makes STLs promising lead compounds in order to develop drugs for cancer treatment.

## 2. Results and Discussion

The phytochemical analyses of some populations and subspecies of *T. vulgare* have shown a remarkable intra-specific variability of chemical constituents due to plant adaptation to habitat conditions. Great evidence of this phenomenon is found in essential oil composition. In fact many chemotypes of *T. vulgare*, collected in different places of Norway [13] and Lithuania [14], have been identified. The non-volatile fraction is also subject to great variability of the terpenoid content both qualitatively and quantitatively. A small plant population native to Bulgaria [15] showed the existence of three pure chemotypes according to the class of STLs detected: no STLs, only germacranolides, only eudesmanolides. Mixed chemotypes are not identified because probably these pure chemotypes are unable to produce hybrids. Similar behavior occurs in Italian *T. vulgare* taxa studied so far [16,17]. In Sicily, apart from the typical form (*T. vulgare* subsp.*vulgare*), a new identified form exists, growing on the eastern side of the Madonie Mountains and on Nebrodi Mountains [18]. This Sicilian subspecies of *T. vulgare* doesn’t show variability of its STLs content as we observed analyzing two separate collections in different years. In fact the results reported below are exactly the same for the two samples.

**Figure 1:**
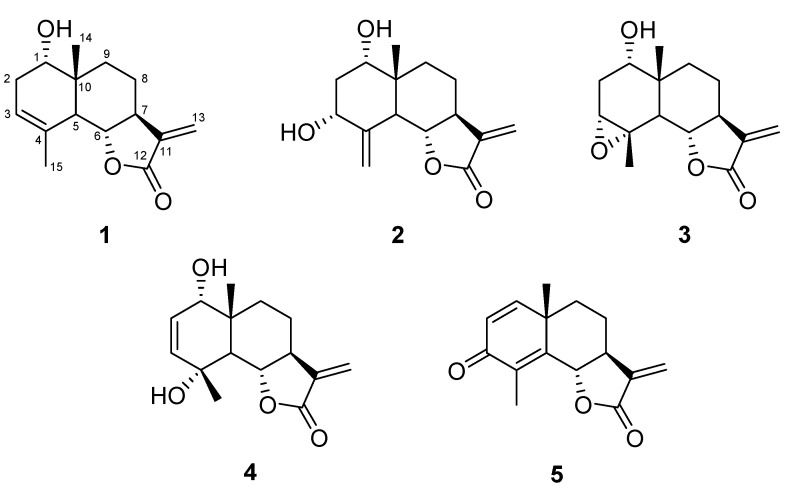
Strucures of compounds **1**–**5**.

The dried flowers of this new subspecies were extracted at room temperature with petroleum ether, in order to eliminate fat substances and successively with CH_2_Cl_2_ and finally with methanol. The dichloromethane extract was purified by repeated silica gel column chromatographies giving five pure compounds identified as STLs belonging all to eudesmanolide class (Figure 1) by comparison of spectroscopic data with literature values. Therefore, this *T. vulgare* species can be assigned to the eudesmanolide chemotype.

In order of polarity, the isolated compounds were: douglanin (**1**), ludovicin B (**2**), ludovicin A (**3**), 1α-hydroxy-1-deoxoarglanine (**4**) and 11,13-dehydrosantonin (**5**), whose structures are shown in Figure 1. All of these products have been previously reported to occur in other plants and in few species of *Tanacetum*. In particular, compound **1** has been discovered in *Artemisia douglasiana* [19] and it has been found in *T. praeteritum* [20] and *T. argenteum* subsp. *canum* [21]. Compounds **2** and **3** have been isolated for the first time in *Artemisia ludoviciana* subsp. *mexicana* [22] and occur in *T. praeteritum* [20]. Compound **4** has been exclusively found in *T. praeteritum* [20]. Finally compound **5** was only isolated from Turkish *Laurus nobilis* [23] and synthetised starting from α-santonin [24,25] but never reported in *Tanacetum* species.

Despite the fact these compounds have been isolated several times from different plant sources, for douglanin (**1**) and ludovicin A (**3**) the ^13^C-NMR data were never reported in literature, and since we consider these data useful for structural elucidation they are described in the Experimental section.

It was recently found that parthenolide, frequently occurring in *Tanacetum* genus and representing the characteristic and active component of *T. partenium*, exerts antiproliferative effects on various cancer cells [26,27]. *T. vulgare* extract also demonstrated activity in the inhibition of mouse leukemia L1210 cells [28]. Furthermore, considering that STLs isolated from *T. praeteritum*, including compounds **1**, **3** and **4**, showed cytotoxic activity against human lung carcinoma cell line GLC4 and the colorectal cancer cell line COLO 320 [29], in our ongoing studies on cytotoxic STLs [30,31,32], we describe herein the effects of the STLs **1**–**5** constituting the dichloromethane extract of the *T. vulgare* subsp. *siculum* flowers against A549 (human lung carcinoma epithelial like) and V79379A (Chinese hamster lung fibroblast like) cell lines.

**Figure 2:**
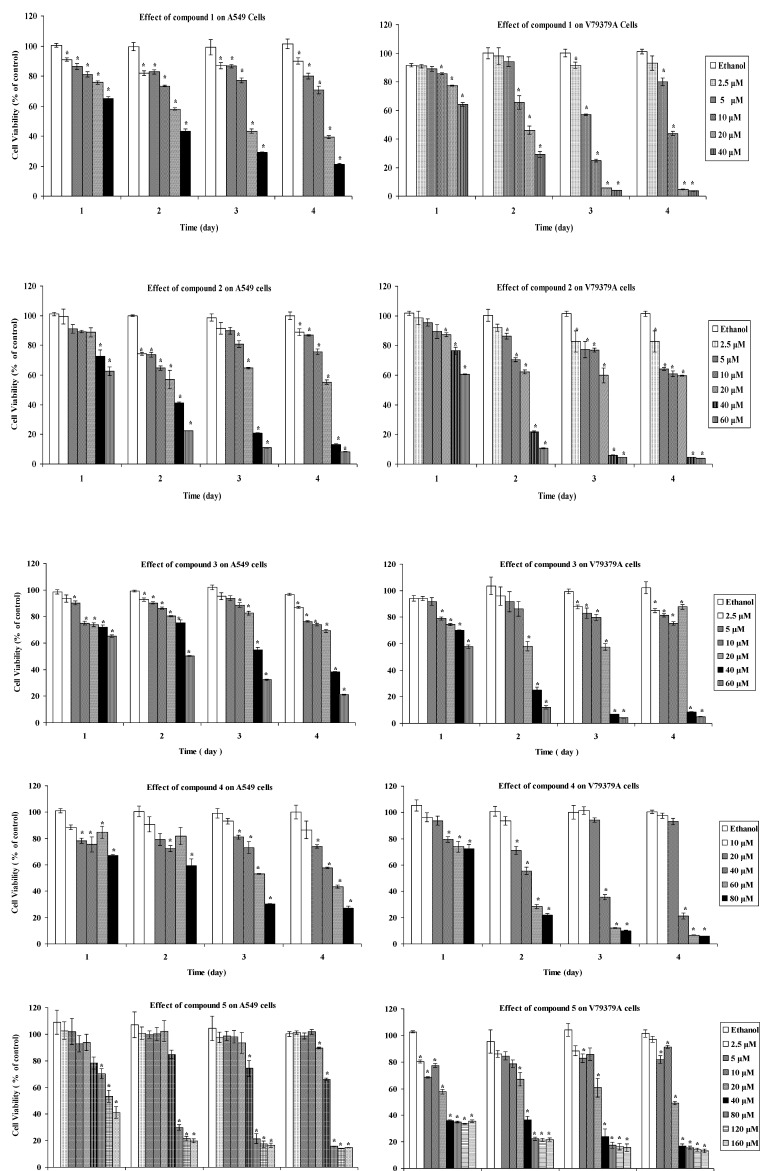
Cell proliferation was measured using MTT colorimetric assay after one, two, three and four day exposures. The results are expressed as the mean ± SD. * Indicates significant difference from the control group by the Tukey test (*p* < 0.05).

The cytotoxic effects were investigated using the tetrazolium salt reduction (MTT; 3-(4,5-dimethylthiazol-2-yl)-2,5-diphenyltetrazolium bromide) assay. The MTT assay is a quick effective method for testing mitochondrial impairment and it correlates well with cell proliferation. Dose-dependent cytotoxicity results, as quantified by the MTT assay for one, two, three, and four days exposures of samples **1**–**5** on A549 and V79379A cells, are shown in the Figure 2 and the IC_50_ values after 3 day exposures are shown in Table 1. The compounds had a generally dose- and time-dependent cytotoxic activity against A549 and V79379A cells. Some solutions of the STLs showed time-and dose-independent cytotoxicity, in fact, the 2.5 μM, 5 μM and 10 μM concentrations of compound **1** showed time-independent effect against A549 cell line for the exposures of four days whereas viability continued to decrease during four days for 20 μM and 40 μM doses. The highest cytotoxic effect of **1** against V79379A was verified for 5 μM and 10 μM doses in a three days exposure, but after four days the cell viability increased. The most effective doses of **1** were 20 μM and 40 μM after three and four days exposures againt A549 cell line, however the same doses were lethal for three and four days exposures against V79379A cells.

**Table 1:** .Cytotoxic activity of tested compounds after three days of exposure (given in IC_50_ values; μM ± SD; n= 3).

Compounds	A549	V79379A
**1**	15.3 ± 0.1	5.0 ± 0.8
**2**	26.4 ± 3.3	23.5 ± 2.2
**3**	34.4 ± 2.4	23.1 ± 1.6
**4**	59.4 ± 3.9	33.4 ± 0.6
**5**	56.6 ± 1.6	26.7 ± 0.9
**cisplatin**	7.7 ± 2.1	32.3 ± 2.5

For both cell lines compound **2** seems to exert a time- and dose-independent response at 2.5 μM, 5 μM and 10 μM concentrations, in fact these solutions were more effective in a two days exposure. Nevertheless 40 μM and 60 μM doses of **2** caused time-dependent cytotoxic activity and they remain active after two, three, four day exposures against A549 cell line and more effectively against V79379A cells.

The 40 μM and 60 μM solutions of **3** decreased the A549 cell viability during four days. The same doses widely effected the V79379A cell viability in three and four days exposures whereas 20 μM concentration showed a time independent behaviour.

Despite the fact that 20 μM and 40 μM solutions of **4** showed more efficacy against the A549 cell line than 60 μM after 1 and 2 days, the 60 μM concentration was more active than 20 μM and 40 μM after 3 and 4 days. Though the 20 μM solution of **4** reduced cell viability of V79379A after two days, an increase of cell viability of V79379A was observed after 3 and 4 days.

The active doses of compound **5** against A549 cell line were higher and in particular 80 μM, 120 μM and 160 μM showed a time dependent cytotoxicity. The doses between 40 μM and 160 μM concentrations of **5** exerted a toxic effect against V79379A cell line.

From the results of this study, we can conclude that the eudesmanolides of *T. vulgare* subsp. *sicilum* (Guss.) are cytotoxic in this test model. Among all, the main compound douglanin (**1**) showed highest level of activity against A549 and V79379A cell lines and compound **4** showed least cytotoxicity, and these results are compatible with earlier published findings [29]. Although compounds **2** and **3** gave close results against V79379A cells, ludovicin B (**2**) is more toxic than ludovicin A (**3**) against A549 cell line according to the IC_50_ values (Table 1). All compounds showed higher levels of activity against the V79379A cell line than against the A549 cell line, in accordance with previous findings [33].

## 3. Experimental

^1^H-NMR spectra were recorded in CDCl_3_ solution on a Bruker Avance DMX300 instrument at 300 MHz, and chemical shifts are reported with respect to the residual CHCl_3_ solvent signal (δ 7.27 ppm). ^13^C-NMR spectra were recorded in CDCl_3_ solution on the same apparatus at 75 MHz, and chemical shifts are reported with respect to the solvent signals (δ_C_ 77.00 ppm). ^13^C-NMR assignments were determined from the DEPT spectra. Optical rotations were measured on a Jasco P-1010 digital polarimeter. MS were recorded on a Shimadzu GCMS QP2010 Ultra system (Kyoto, Japan). Elemental analysis was carried out with a Perkin-Elmer 240 apparatus (Waltham, MA, USA). Merck Si gel (70–230 mesh; Darmstadt, Germany), deactivated with 15% H_2_O (w/w), was used for column chromatography.

### 3.1. Plant Material

The flowers of *Tanacetum vulgare* subsp. *siculum* (Guss.) Raimondo et Spadaro, were collected from blooming plants at Caserma Mafauda ( 1,250 m a.s.l.), Nebrodi Mountians, Sicily, in July 2010. Samples of the studied material, identified by F. M. Raimondo and V. Spadaro, are kept in the Herbarium Mediterraneum of the Palermo University [Raimondo & Spadaro (PAL)].

### 3.2. Extraction and Isolation

Dried and finely powdered flowers of *T. vulgare* subsp. *siculum* (340 g) were sequentially extracted by cold maceration with petroleum ether (b.p. 40–60 °C, 3 × 2.5 L) and sequentially with dichloromethane (3 × 2.5 L). After filtration, the dichloromethane was evaporated at low temperature (35 °C) yielding a gum (16 g) which was chromatographed over a silica gel dry column with a solvent gradient from 100% petroleum ether (b.p. 40–60 °C) to 100% EtOAc. The fraction eluted with petroleum ether/EtOAc (2:1) yielded douglanin (**1**) (350 mg). The fraction eluted with petroleum ether/EtOAc (3:2), was further purified, by column chromatography, with petroleum ether/EtOAc (7:3) as eluent to afford, in order of increasing polarity, ludovicin B (**2**, 120 mg), ludovicin A (**3**, 20 mg), 1α-hydroxy-1-deoxoarglanine (**4**, 50 mg), 11,13-dehydrosantonin (**5**, 10 mg).

*Douglanin* (**1**): data previously reported [19]. ^13^C-NMR (CDCl_3_) *δ*: 16.8 (q, C-14), 20.6 (t, C-8), 23.3 (q, C-15), 31.5 (t, C-2), 32.6 (t, C-9), 40.1 (s, C-10), 43.9 (d, C-5), 50.4 (d, C-7), 72.0 (d, C-1), 81.8 (d, C-6), 116.3 (t, C-13), 118.8 (d, C-3), 132.7 (s, C-4), 138.6 (s, C-11), 170.8 (s, C-12).

*Ludovicin B* (**2**): lit. [34].

*Ludovicin* A (**3**): data previously reported [22]. ^13^C-NMR (CDCl_3_) *δ*: 18.1 (q, C-14), 21.0 (t, C-8), 22.3 (q, C-15), 29.1 (t, C-2), 33.1 (t, C-9), 40.1 (s, C-10), 46.0 (d, C-5), 50.8 (d, C-7), 59.0 (s, C-4), 60.8 (d, C-3), 73.2 (d, C-1), 81.0 (d, C-6), 117.2 (t, C-13), 138.5 (s, C-11), 170.4 (s, C-12).

1α-Hydroxy-1-deoxoarglanine (**4**): lit. [20].

11,13-Dehydrosantonin (**5**): lit. [24,25].

### 3.3. Cell Cultures

The cytotoxicity of the STLs was measured against A549, human lung carcinoma epithelial-like, and V79379A, Chinese hamster lung fibroblast-like, cell lines obtained from Institute for Fermentation, Osaka (IFO, Osaka, Japan). A549 cell line was maintained as a monolayer in RPMI 1640 Culture Medium (Gibco, Grand Island, NY, USA) containing 10% (v/v) heat-inactivated Fetal Bovine Serum (Sigma, Steinheim, Germany), Penicillin-Streptomycin (Sigma) and sodium bicarbonate. V79379A cell line was maintained as a monolayer in Dulbecco’s Modified Eagle Medium-High Glucose (DMEM) (Sigma) containing 10% (v/v) heat-inactivated Fetal Bovine Serum (Sigma), Penicillin-Streptomycin (Sigma) and sodium bicarbonate. A549 and V79379A cells were plated onto 25 cm^2^ tissue culture flasks (TPP, Europe, Trasadingen, Switzerland) and incubated at 37 °C in a humidified atmosphere of 5% (v/v) CO_2_ in air for 24 h. Stock solutions of the compounds were initially prepared in ethanol absolute (Riedel de Haen, Hannover, Germany) and further diluted in fresh complete medium. The concentration of ethanol in tissue culture plates was lower than 0.1% (v/v) in all experiments.

### 3.4. Cytotoxicity Assay

The growth inhibitory effects of the compounds were measured using the 3-(4,5-dimethylthiazol-2-yl)-2,5-diphenyltetrazolium bromide (MTT) assay [35]. After A549 cells were seeded in 96-multiwell tissue culture test plates (TPP) at a density of 5,000 cells/well, V79379A cells were seeded in 96-multiwell plates (TPP) at a density of 1,000 cells/well. After a 24-h pre-incubation period, the medium was discarded and replaced with different concentrations of the freshly prepared test compounds in complete medium. Negative control groups were untreated cells and positive control groups were the ones treated with pure ethanol. 

In the positive control groups, ethanol was added to obtain the final concentration of max. 0.1%. Just before the experiments, stock solutions were diluted with the supplemented mediums to obtain final concentrations of 0–100 μM. Cisplatin [*cis*-dichlorodiamine-platinum(II), Sigma] was dissolved in DMSO max. 0.1% (Merck, Europe, Darmstadt, Germany) immediately before use, and it’s IC_50_ value was tested to compare with sesquiterpene lactones activity against cell lines. These plates were then incubated for 1, 2, 3 and 4 days, respectively, before the viability of cells was determined by MTT assay. After incubated period exposure of either of the samples containing medium from each well, the medium was then replaced with 100 μL fresh medium containing 0.5 mg/mL MTT (Sigma) dissolved in phosphate buffer saline (PBS). The plates with added MTT solution were then wrapped in aluminium foil and replaced in the 5% CO_2_ incubator for 2 h. At the end of this period, the medium was removed and the formazan crystals formed by MTT metabolism were dissolved by addition of 100 μL DMSO (Merck) to each well. Then, the plates were gently mixed on a plate shaker approximately for 5 min, and their absorbances were read at 570 nm with a microtiter plate reader (Bio-Tek, ELX808IU, Winooski, VT, USA). All experiments were repeated at least three times. The SPSS software was used for the statistical analyses of assessment of the MTT assay. Data were evaluated using one-way ANOVA followed by the Tukey test. A value of *p* < 0.05 was considered significant. Homemade software “Helper of Cell Culture Lab. v.1” created by Mehmet Varol was used for calculation of IC_50_ values, the software was tested with GraFit Data Analysis Software version 6.

## 4. Conclusions

The studied subspecies of *T. vulgare* showed no chemical composition variability and it can be classified in belonging to the eudesmanolide chemotype. All compounds had cytotoxic activity against *in vitro* cultured cancer and healthy cell lines. However, these products could not be useful for medical treatments against tumour cells because of their higher activity against healthy cells (Table 1). Although cisplatin, frequently used as an anti-cancer drug, is more active against the A549 cancer cell line (IC_50_ = 7.7 ± 2.1) than against V79379A healthy cell line (IC_50_ = 32.3 ± 2.5), the sesquiterpene lactones of *T. vulgare* subsp.*sicilum* (Guss.) are found more effective against the V79379A healthy cell line than A549 tumour cells.
